# Single-Cell RNA-Seq Analysis Reveals Macrophage Involved in the Progression of Human Intervertebral Disc Degeneration

**DOI:** 10.3389/fcell.2021.833420

**Published:** 2022-02-28

**Authors:** Zemin Ling, Yong Liu, Zhe Wang, Ziji Zhang, Bolin Chen, Jiaming Yang, Baozhu Zeng, Yu Gao, Chang Jiang, Yulin Huang, Xuenong Zou, Xiuhui Wang, Fuxin Wei

**Affiliations:** ^1^ Guangdong Provincial Key Laboratory of Orthopedics and Traumatology, Department of Spinal Surgery, The First Affiliated Hospital of Sun Yat-sen University, Guangzhou, China; ^2^ Department of Joint Surgery, The First Affiliated Hospital of Sun Yat-sen University, Guangzhou, China; ^3^ Department of Orthopedics, Zhongshan Hospital, Fudan University, Shanghai, China; ^4^ Department of Orthopedics, The Seventh Affiliated Hospital of Sun Yat-sen University, Shenzhen, China; ^5^ Department of Orthopaedics, Shanghai University of Medicine and Health Sciences Affiliated Zhoupu Hospital, Shanghai, China

**Keywords:** intervertebral disc degeneration, single-cell RNA sequencing, nucleus pulposus, gene, inflammation, metabolism

## Abstract

Intervertebral disc degeneration (IDD) has been considered as the primary pathological mechanism that underlies low back pain. Understanding the molecular mechanisms underlying human IDD is imperative for making strategies to treat IDD-related diseases. Herein, we report the molecular programs, lineage progression patterns, and paths of cellular communications during the progression of IDD using single-cell RNA sequencing (scRNA-seq) on nucleus pulposus (NP) cells from patients with different grades of IDD undergoing discectomy. New subtypes of cells and cell-type-specific gene signatures of the metabolic homeostatic NP cells (Met NPC), adhesive NP cells (Adh NPC), inflammatory response NP cells (IR NPC), endoplasmic reticulum stress NP cells (ERS NPC), fibrocartilaginous NP cells (Fc NPC), and CD70 and CD82^+^ progenitor NP cells (Pro NPC) were identified. In the late stage of IDD, the IR NPC and Fc NPC account for a large proportion of NPC. Importantly, immune cells including macrophages, T cells, myeloid progenitors, and neutrophils were also identified, and further analysis showed that significant intercellular interaction between macrophages and Pro NPC occurred *via* MIF (macrophage migration inhibitory factor) and NF-kB signaling pathways during the progression of IDD. In addition, dynamic polarization of macrophage M1 and M2 cell subtypes was found in the progression of IDD, and gene set functional enrichment analysis suggested a significant role of the macrophage polarization in regulating cell metabolism, especially the Pro NPC. Finally, we found that the NP cells in the late degenerative stage were mainly composed of the cell types related to inflammatory and endoplasmic reticulum (ER) response, and fibrocartilaginous activity. Our results provided new insights into the identification of NP cell populations at single-cell resolution and at the relatively whole-transcriptome scale, accompanied by cellular communications between immune cells and NP cells, and discriminative markers in relation to specific cell subsets. These new findings present clues for effective and functional manipulation of human IDD-related bioremediation and healthcare.

## Introduction

Low back pain (LBP), as one of the most common disorders in the musculoskeletal system (Ferguson and Steffen, 2003; Paige et al., 2017; Tong et al., 2017), causes significant economic losses worldwide each year (Hoffman and Dow, 2016; Juch et al., 2017; Hall et al., 2019). It has been reported that about 80% of adults suffered from low back or neck pain at least once during their lifetime, and the majority of patients would lose their labor capacity (Dawson et al., 2011; Blanquer et al., 2015; Sakai and Andersson, 2015). Despite the improvement in surgery and medicine, LBP remains one of the main causes of health expenditure and financial burden.

Intervertebral disc degeneration (IDD) has been considered as the primary pathological mechanism that underlies LBP, and receives increasing attention from precision-related regenerative medicine (Zingg and Kendall, 2017). It has been reported that the initial degeneration of intervertebral disc may be presented as early as in the adolescence, when 20% of youngsters have mild signs of the IDD (Boxberger et al., 2009). The disc is a physiological non-self-renewing avascular tissue and consists of annulus fibrosus and nucleus pulposus (NP), which undergoes cellular and extracellular matrix (ECM) changes during IDD at the early pathological stage (Mwale et al., 2014). The process of IDD encompasses the structural damage of the disc and the changes in number and composition of cells. With aging and advancing degeneration, the NP is primarily affected (Kos et al., 2019). Due to the poor self-repair ability of NP, it is difficult to reverse the degenerative process. Thus, it is still a challenge to repair or regenerate the structure and functions of the intervertebral disc by conservative or surgical therapies (Yim et al., 2014; Wang et al., 2015).

Although many researches (Risbud and Shapiro, 2014; Vergroesen et al., 2015) had shed light on identifying the various factors responsible for disc degeneration (inflammation, apoptosis, immunity, senescence, etc.), as well as strategies for regeneration, effective measures that lend promise in the coming years are still lacking. A better understanding of the pathophysiology and the mechanisms underlying the role of NP in the process leading to IDD is of critical importance. Nevertheless, recent biological approaches have gained thrust in the field of IDD and intense efforts have focused on the gene therapy or endogenous repair for NP regeneration in treating IDD (Sampara et al., 2018; Ma et al., 2019; He et al., 2021). However, NP cell-type composition, biochemical markers that can effectively predict IDD, and the cellular heterogeneity leading to IDD progression remain largely unknown.

Single-cell RNA sequencing (scRNA-seq) of different tissues has provided us an option to map the types, subsets, and states of cells in healthy and unhealthy conditions in an unprecedented manner (Sharma et al., 2018; Szabo et al., 2019; Iyer et al., 2020), which can also be used to provide deep insights into physiological and pathological processes (Svensson et al., 2017; Tian et al., 2019). For instance, Tang et al. recently reported that they identified seven molecularly defined populations of chondrocytes in the human osteoarthritis (OA) cartilage, and clarified the role of different cell types for the early diagnosis and treatment of OA (Ji et al., 2019). Zhang et al. used scRNA-seq to identify the cell subsets and their gene signatures in healthy human and degenerated meniscus cells (Sun et al., 2020), which could provide new insights into cell-based tissue engineering and novel therapeutic strategies. The development of scRNA-seq provides us with a new strategy for unprecedentedly deep data mining based on the level of single-cell gene expression and interaction, which would definitely shed light on novel cell therapies for multiple human diseases.

Here, we used scRNA-seq to chart a comprehensive census of NP cells from different grades of degenerated intervertebral discs based on the Pfirrmann grading system (Pfirrmann et al., 2001). We identified various cell subsets and their gene signatures to determine their differentiation relationships and characterize diversity within specific cell types. We also demonstrated the existence of NP stem/progenitor cells and their corresponding marker genes, and the interactions between immune cell types and NP progenitor cells and other NP cell types *via* cell communication analysis. Finally, we investigated the integral influence of IDD on NP cellular heterogeneity and identified a potential therapeutic target for IDD-related diseases.

## Results

### Single-Cell Transcriptomic Overview and Clustering of the Human NP Tissue

**FIGURE 1:**
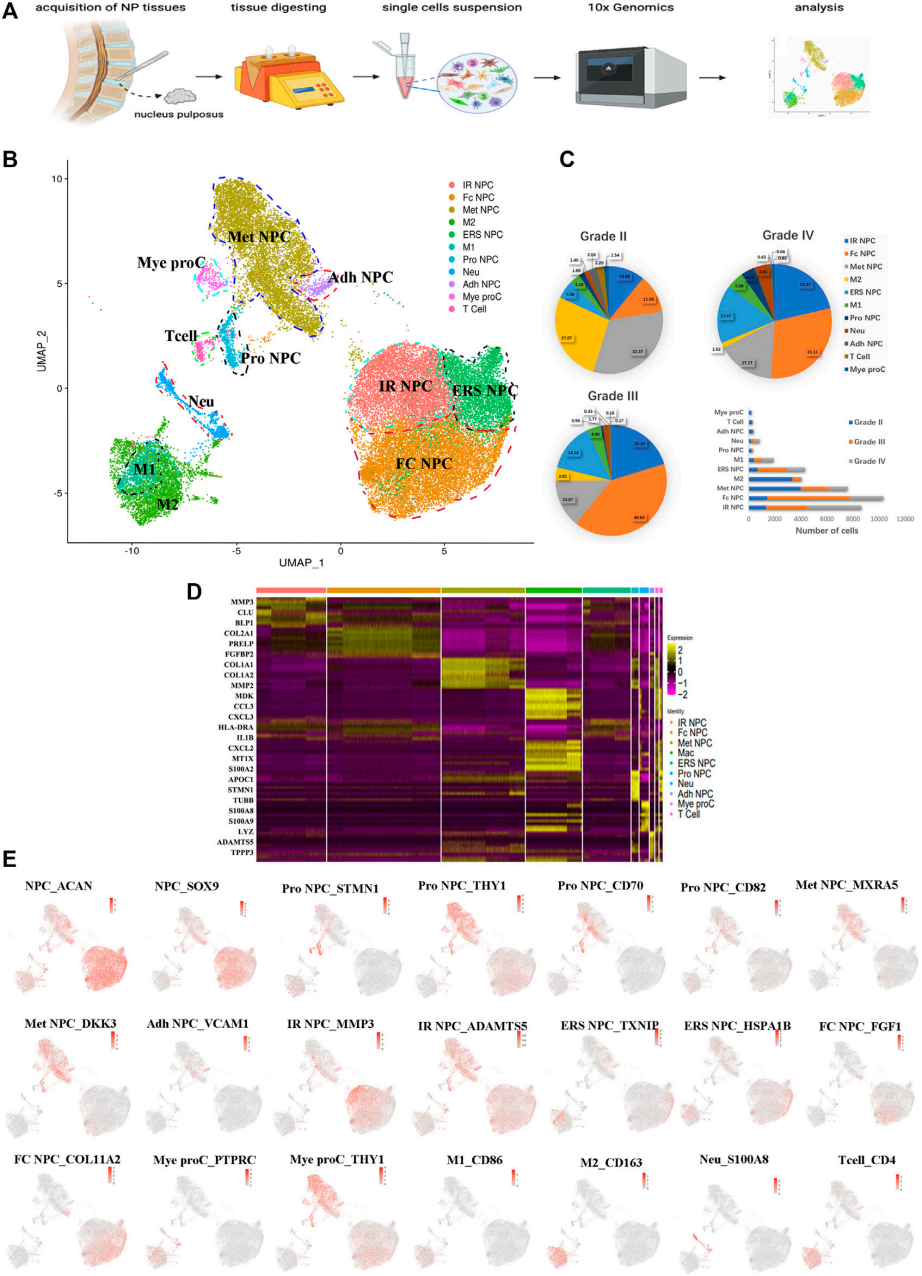
A single-cell atlas of human nucleus pulposus (NP) with IDD. **(A)** Schematic workflow of this experiment. **(B)** Uniform manifold approximation and projection (UMAP) view of 36,196 single cells, color-coded by assigned cell type. **(C)** The pie charts and bar plot depicting the proportions of cell type within different grading of NP tissues. **(D)** Heatmap revealing the scaled expression of differentially expressed genes for each cell cluster. **(E)** Feature plot map showing the expression levels and distribution of representative marker genes of each cell cluster on the UMAP map.

To understand the clustering change and the molecular features of human NP cells during IDD, we disassociated NP tissues from three human IDD patients with different Pfirrmann degenerative grades [Pfirrmann II (between II and III, but more close to II), III, and IV], and single-cell RNA-seq of cells (scRNA) was performed based on the 10X Genomics platform (Figure 1A). There were 36,196 cells that had been successfully isolated from NP cell suspensions. The heterogeneity of cells was classified by gene expression and the functional analysis, and scRNA sequencing of NP tissues identified 11 clear separations of cell types, which included six types of NP cells (expressing SOX9 and ACAN) and five types of immune cells (Figure 1B). The proportion of each cell cluster in Pfirrmann grades II, III, and IV NP tissues is shown in Figure 1C. The heterogeneity of NPCs was classified by gene expression (Figure 1D), and the representative gene markers are shown in Figure 1E with the feature plot map. For instance, the collage 2A1 (col2A1), aggrecan (ACAN), and SOX9 were highly expressed in NP cells (Tu et al., 2021). Subsequently, six subtypes of NP cells, namely, metabolic homeostatic NP cells [Met NPC, highly expressing MXRA5 and DKK3 (Li et al., 2017; Poveda et al., 2017)], adhesive NP cells [Adh NPC, highly expressing VCAM1 (Rafat et al., 2012)], inflammatory response NP cells (IR NPC, highly expressing MMP3 and ADAMTS5), endoplasmic reticulum stress NP cells [ERS NPC, highly expressing TXNIP and HSPA1B (Ding et al., 2020)], fibrocartilagenous NP cells [Fc NPC, highly expressing FGF1 (Fernández et al., 2010)], and CD70^+^ and CD82^+^ progenitor NP cells (Pro NPC), were identified. As the proportion alterations of 11 cell types in grades II, III and IV are shown in Figure 1C, we can find that the proportion of metabolic homeostatic NP cells (Met NPC, 32.37%) was the highest at the early period (grade II) of disc degeneration, then the proportion of fibrocartilagenous NP cells (Fc NPC) increased at the middle (grade III, 40.64%) and late (grade IV, 31.11%) period of disc.

In addition, immune cells including macrophages (M1 and M2), T cells, myeloid progenitors, and neutrophils have also been found in the NP tissues. The clusters of immune cells were enriched with genes involved in immune response and inflammatory response, confirming the cell populations [like CD68^+^ and CD163^+^ for macrophage (Evans et al., 2013; Alvarado-Vazquez et al., 2017), S100A8^+^ for neutrophil] (Figure 1E). Furthermore, the results showed that the numbers of inflammatory response NP cells increased in grade IV than in grade II, but the metabolic homeostasis NP cells were significantly decreased, suggesting that NP cells may be influenced by the inflammatory factors from keeping homeostasis during disc degeneration. With the progression of IDD, the M2 polarized macrophage decreased significantly, followed by the cluster of progenitor NPC (Pro NPC). In contrast to M2, the proportion of M1 polarized macrophage increased gradually (Figure 1C), suggesting that the activity of Pro NPCs may be influenced by the macrophage polarization during disc degeneration.

### Phenotype of NP Cells was Dramatically Altered in Different Grade IDD Patients

**FIGURE 2:**
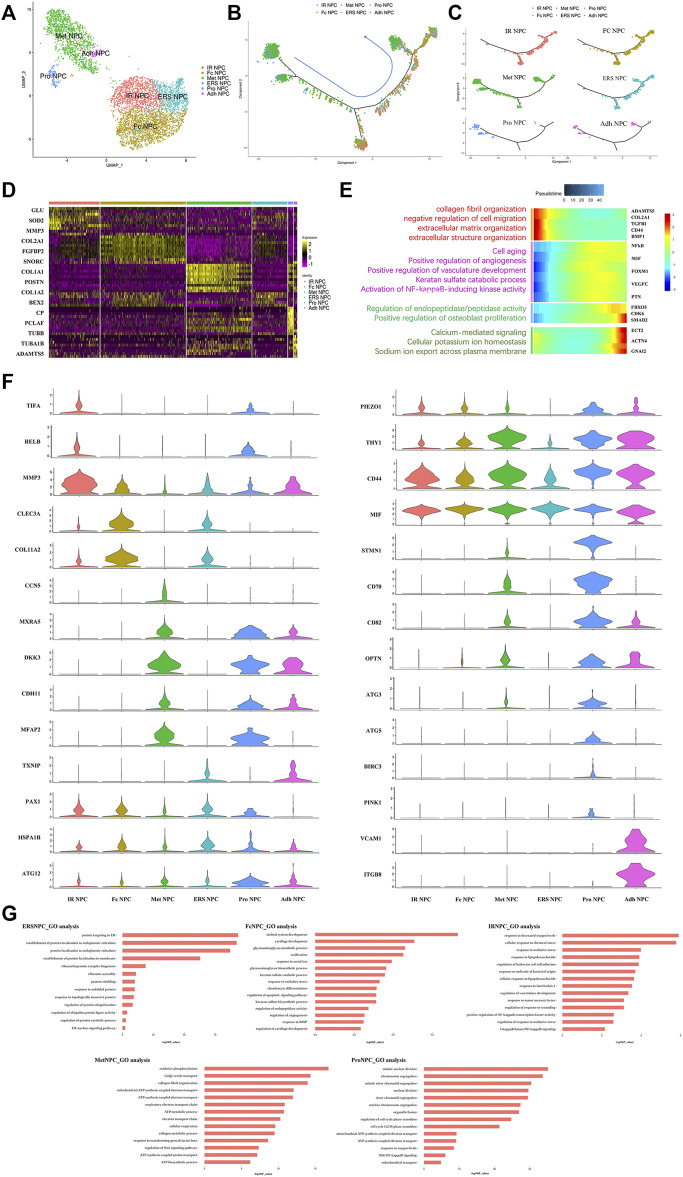
Single-cell RNA sequencing revealed transcriptional features of NPC subclusters. **(A)** UMAP view of NP cells, color-coded by subtypes **(B,C)** Monocle pseudotime trajectory showing the progression of Pro NPC, Met NPC, Adh NPC, IR NPC, FC NPC, and ERS NPC. **(D)** Heatmap revealing the scaled expression of differentially expressed genes for each NPC cluster. **(E)** The heatmap of genetic change with pseudotime and gene functional enrichment analysis of these genes in the progression of IDD. **(F)** The violin plot map of typical expressed genes in each subcluster of the NPCs. **(G)** The Gene Ontology (GO) analysis of each cluster of NPCs.

To our knowledge, insufficient nutrients were one cause of halted cell cycle and increased apoptosis in proliferating cells, and we had reported that endplate sclerosis may hinder nutrient penetration to NP tissue (Ling et al., 2020), which may induce IDD. To discover the proportion and gene changes of NP cells during disc degeneration, the NP cells were subsequently divided into six subtypes based on marker gene and the gene functional enrichment analysis (Figures 2A,G). We found that three subtype clusters, namely, IR NPC, ERS NPC, and Fc NPC, had been divided from inflammation NPC. What is more, to explore the gene changes of NPCs and identify novel markers for different cell types, the developmental trajectory of NP cells had been done, which revealed the cluster of Pro NPCs had proliferative activities at the early stage (Figure 2B). Similar to Adh NPCs, the Met NPCs were mainly expressed in early and late stages. However, IR NPCs, ERS NPCs, and Fc NPCs all had a similar trend by trajectory analysis (Figure 2C), which may mean a similar fate for them. With the degeneration of nucleus pulposus cells, the gene expression pattern changed (Figure 2E). We found that the expressions of NFkB and MIF increased gradually over time, and gene functional enrichment analysis also showed that the NFkB signaling pathway was activated at the late stage of degeneration, these suggested that there was a possible association between NFkB and MIF.

We next performed Gene Set Functional Enrichment Analysis to evaluate the molecular differences among the six types of NP clusters (Figure 2D). The results of the Heatmap (Figure 2F) indicated that DKK3 and MXRA5, which were related with transforming growth factor-β (TGF-β) signaling pathway (Li et al., 2017; Poveda et al., 2017), were upregulated in Met NPC. This predicted that TGF-β signaling pathway might play an important role in metabolism of NPCs. DDK3 and MXRA5 were also upregulated in Pro NPC and Adh NPC, suggesting that the cell metabolic homeostasis exists in different cell subtypes during disc degeneration. The genes of OPTN, ATG3, ATG5, and ATG12, which were related to the autophagy of mitochondria (Hailey et al., 2010; Radoshevich and Debnath, 2011; Richter et al., 2016), were significantly upregulated in Pro NPCs. This suggested mitochondrial autophagy might be important for keeping activity of Pro NPC. In addition, CD70 and CD82 were also significantly upregulated in Pro NPC, which might be the potential cell marker of this cluster. Importantly, macrophage migration inhibitory factor (MIF) was significantly upregulated in the six subclusters of NPCs, especially in Met NPC and Pro NPC. A previous study reported that MIF played an important role in macrophage activation (Kim et al., 2020). This predicted that MIF-mediated macrophage polarization might play an important role in the cellular activity of Pro NPC.

### Phenotype of Immune Cells in NP Tissue was Altered in Different Grade IDD Patients

**FIGURE 3:**
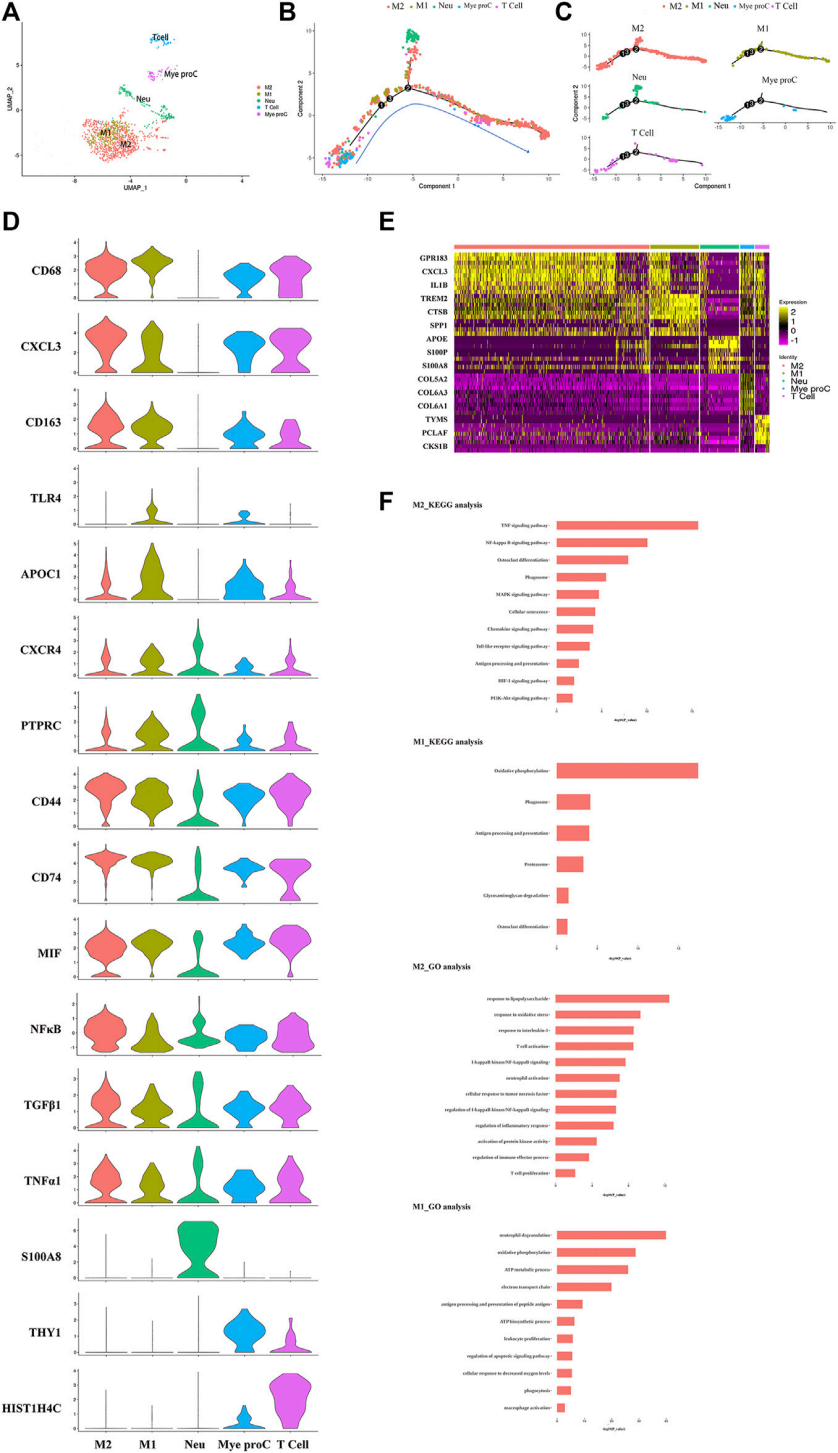
Single-cell RNA sequencing revealed transcriptional features of immune cell clusters. **(A)** UMAP view of the immune cells, color-coded by subtypes. **(B,C)** Monocle pseudotime trajectory showing the progression of the immune cells. **(D)** The violin plot map of typical expressed genes in each subcluster of the immune cells. **(E)** Heatmap revealing the scaled expression of differentially expressed genes for each immune cell cluster. **(F)** The Gene Ontology (GO) and Kyoto Encyclopedia of Genes and Genomes (KEGG) analysis of M1 and M2 polarization of macrophage cluster.

The immune cells including macrophages, T cells, myeloid progenitor cells, and neutrophils, were also been identified in the NP tissues. After the functional analysis, the results showed that most of the cells were involved in neutrophil activation, neutrophil degranulation, and regulation of the apoptotic signaling pathway (Figures 3A,F). Although there were a large proportion of the M1 macrophage and M2 macrophage in immune cells, most myeloid progenitors and T cells and a certain proportion of M2 macrophage populated at the root of the trajectory (Figure 3B), which indicated that the immune cells especially M1 macrophage and M2 macrophage contribute to all periods of disc degeneration (Figure 3C). As we know, macrophage polarization involved in inflammatory cascade might contribute to the process of IDD (Ling et al., 2020). To determine which gene affects macrophage cell polarization during immunization with the progression of IDD, functional prediction and the gene expression analysis of each cell cluster had been done (Figures 3D,E). The results carried out using heatmap showed that their genes being upregulated (MIF in both M1 and M2, CXCL3 and SELENOP in M2 macrophage, SPP1 and APOC1 in M1 macrophage, and S100A8 and S100A9 in neutrophil) might play a crucial role in the cellular activity and polarization. An interesting finding is that the proportion of M2 macrophage cluster drops with the progression of IDD, followed by the increasing trend of the proportion of M1 macrophage (Figure 1C). This suggested that macrophage polarization might play an important role in amplifying the inflammatory cascade in the process of IDD. The cellular interaction analysis among the immune cells also showed that nuclear factor kappa B (NF-κB) was highly expressed in both M1 and M2 macrophage, which suggested that NF-κB also played an important role in the macrophage polarization in the NP tissue.

### Cell-to-Cell Communication Between NP Cells and Immune Cells in NP Tissue

**FIGURE 4:**
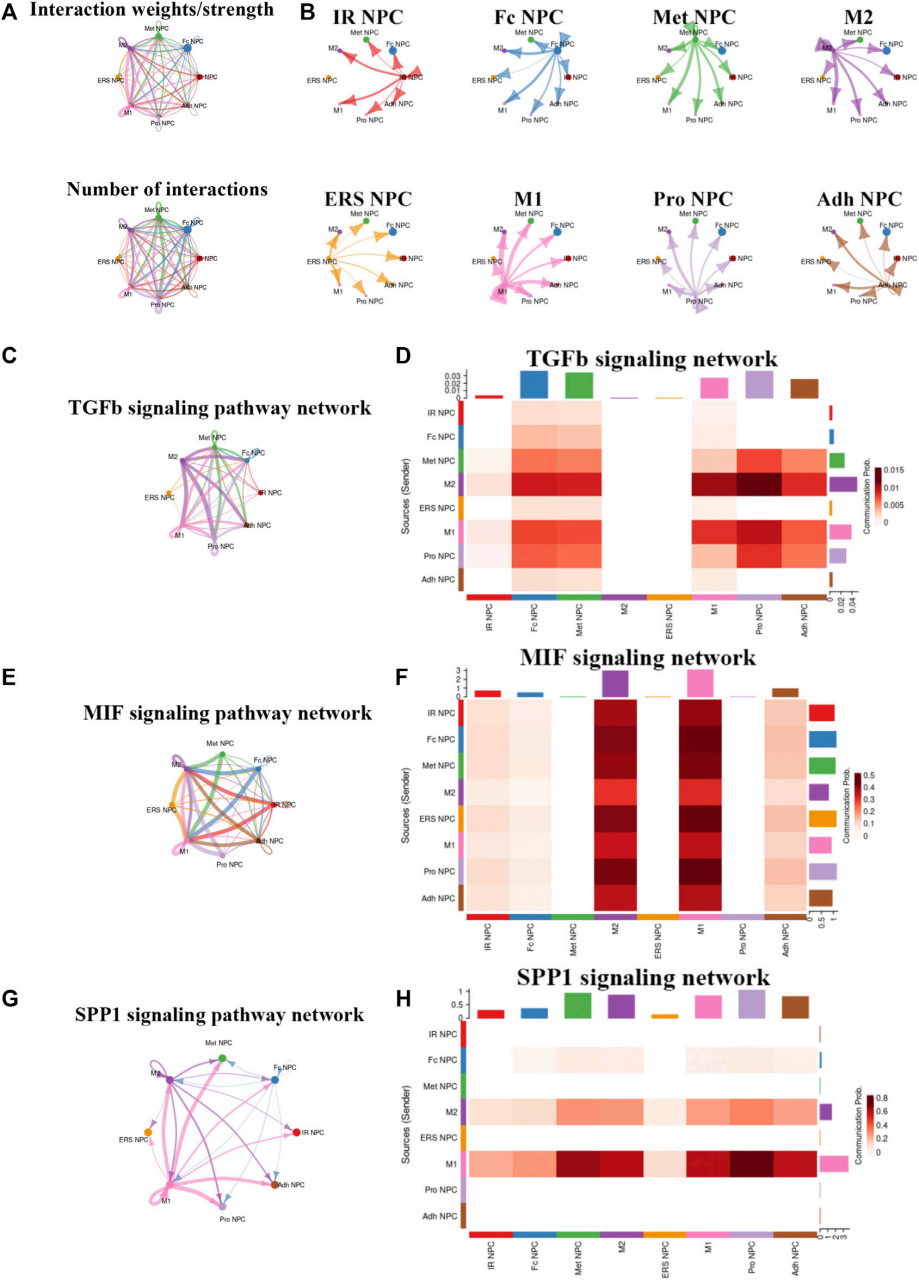
Cellular interaction analysis by CellChat. **(A,B)** The cellular interaction weights and number of interactions between the major clusters of NPCs and macrophage. **(C,D)** The TGF-beta signaling pathway network was significantly detected between the interactions of Macrophage and Pro NPCs. **(E,F)** The macrophage migration inhibitory factor (MIF) signaling pathway network was significantly detected between the interactions of macrophage and each NPC cluster. **(G,H)** The secreted phosphoprotein 1 (SPP1) signaling pathway network was significantly detected between the interactions of M1 polarized macrophage and Pro NPC, Met NPC and Adh NPC.

**FIGURE 5:**
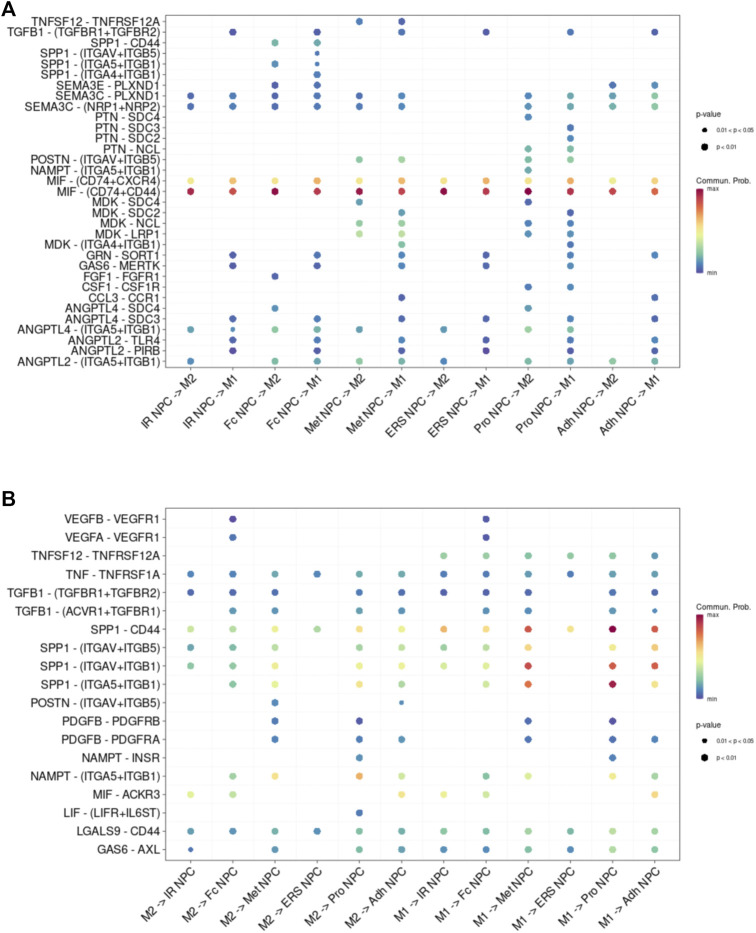
Cell–cell interaction networks in macrophage and each cluster of NPCs. **(A)** The cell–cell interaction network of NPC cluster on macrophage. **(B)** The cell–cell interaction network of macrophage on NPC clusters.

To study the main factors and the pathways that contribute to activation and steady state of NP homeostasis, especially between NP cells and immune cells under insufficient nutrient and inflammatory cascade in the process of IDD, CellChat analysis was performed to assess cell-to-cell communication in the whole cell populations. From the results of visualization inferred cell–cell communication network of the number of interaction and interaction weights (Figures 4A,B), strong cell interactions were found not only between M1 and M2 macrophages, among different kinds of NP cells, but also from the macrophages to all kinds of NP cells. Additionally, to uncover the pathway changes of different subtype clusters of NP tissue, significant ligand–receptor interactions including outcoming (Figure 5A) and incoming (Figure 5B) pattern of secreted signaling cellular communication among cells in NP tissue had been done; the MIF pathway and Secreted Phosphoprotein 1 (SPP1) pathway had a significant change between immune cells and NP cells (Figures 4E–H), especially between macrophages and Pro NPCs. As MIF was highly expressed in the cell clusters of MAC M1, Pro NPC, and IR NPC, cellular interaction analysis also showed that the MIF signaling pathway was highly activated between M1 and Pro NPC, ERS NPC, and Fc NPC (Figures 4E,F). The TGF-β pathway had been reported to be involved in the regulation of NP cell (Ling et al., 2020). In addition, in the TGF-β signaling network, M2 macrophage had connections to all kinds of NPC except for ERS NPC, and especially had strong connections to M1 macrophage and pro NPC (Figures 4C,D). These data indicated that M2 macrophage might be involved not only in regulating the macrophage polarization with M1 but also in regulating the function of Pro NPC by MIF and TGFβ signaling networks.

**FIGURE 6:**
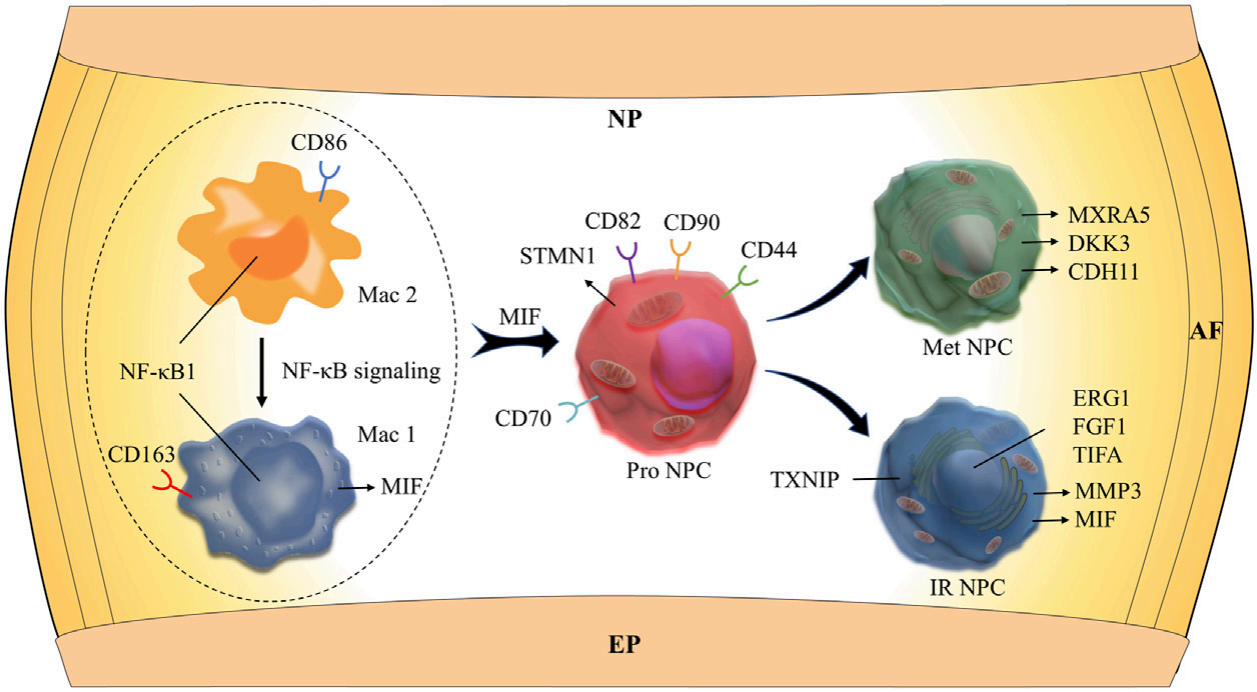
Schematic graph of the potential hypothetical cellular interactions and pathways.

The functions of these subsets remained to be elucidated. These findings provided a point of reference for examining the role of NP cell and immune cell subsets in the NP tissue, especially the potential regulatory mechanism between the immune cells and NP cells.

## Discussion

In this study, we used scRNA-seq to chart a comprehensive census of NP cells from different grades of intervertebral disc. We identified 11 clear separations of cell types, which included six types of NP cells and five types of immune cells. Then, the cellular communications, discriminative markers, and transcription factors in relation to specific cell subsets (i.e., NP cells and immune cells) were also analyzed. The results showed that the dynamic polarization of macrophage M1 and M2 cell subtypes, which might be mediated by NF-kB signaling pathways, plays a crucial role in the activity of NP progenitor cells. These new findings present clues for effective and functional manipulation of human IDD-related bioremediation and healthcare.

IDD and the related degenerative disc disease (DDD) have been recognized as a major cause of lower back pain. Although numerous methods are employed for the treatment of DDD, including physical therapy in the early stages and spinal fusion in the more advanced cases (Dowdell et al., 2017), all these therapies do not prevent DDD from progression. Moreover, spinal fusion surgery is a cost-effective procedure undertaken to relieve pain and restore spinal function for symptomatic DDD (Kim et al., 2018; Overley et al., 2018). An increasingly aging society, economic pressure, and the DDD epidemic all emphasize the demand for new strategies for the diagnosis and treatment of early-stage DDD, ultimately to reduce the need for spinal fusion surgery (Liu et al., 2020). Currently, an increasing number of studies support the idea that gene- and cell-based strategies effectively alleviate disc degeneration and improve regeneration (Oehme et al., 2015; Vedicherla and Buckley, 2017; Chen et al., 2020; Takeoka et al., 2020). Although effective outcomes have been reported by many *in vivo* studies (Feng et al., 2017; Gan et al., 2017), their clinical translation is significantly hindered due to the different cell types between species and less detailed understanding of the internal state of NP cell in IDD pathogenesis (McCann and Séguin, 2016). Thus, a deep elucidation of the gene- and cell-based pathogenesis of IDD is urgently needed for the promising biological intervention in the near future.

Herein, we investigated the NP cells at different stages at single-cell resolution using comprehensive gene expression profiling. To our knowledge, this is one of the earliest studies to describe the transcriptional cell atlas of NP tissues at single-cell resolution. We identified novel cell markers and signatures to verify each hypothesized NP cell cluster. It is worth noting that, besides the empirically inferred NP subtypes, we identified several new subtypes of NP cells, potential markers of the NP cell populations with strong proliferative ability, and the signaling pathways involved in the cell interaction and IDD pathogenesis based on scRNA-seq analysis. Importantly, we identified the CD70^+^ and CD82^+^ NP progenitor cells with highly proliferative ability and found the intimate interactions between macrophage cell types and NP progenitor cell types based on the single-cell resolution. We also found that the NP cells in the late degenerative stage are mainly composed of the cell types related to inflammatory and endoplasmic reticulum (ER) response, and fibrocartilaginous activity. These findings are exciting for us, as we exploit new strategies that contribute to the early diagnosis and therapeutic treatment of human DDD.

The matrix of the intervertebral disc consists mainly of a fibrillar collagen network that offers tensile strength (Antoniou et al., 1996) and aggregates proteoglycans that resist compressive loading. These major components form a mesh suited for containing water molecules, especially in the nucleus. The collagens of the disc comprise two major collagens: type I and II, which are the major constituents of the nucleus pulposus and annulus fibrosus, respectively (Eyre and Muir, 1976). In our scRNA-seq results, new subtypes of NP cells (Fc NPC) highly expressing collagen type II alpha 1 (col2A1) were found in the late stage of IDD process. Moreover, the proportion of this cell cluster showed an increasing trend with the progression of IDD. It seems that this result is not consistent with the previous studies, which revealed that the content of type II collagen decreased accompanied by type I collagen and increased with aging and the progression of IDD (Kim et al., 2003; Wei et al., 2015). This might be due to the debilitating synthesis of type II collagen with development and aging. It has been reported that the denaturation of type II collagen in discs, independent of grade, from the 2- to 5-year age group was markedly higher than that from the 0- to 2-year age group [48]. This high level of denaturation seen in the juvenile was also observed when degeneration developed in the adult (Antoniou et al., 1996). Thus, improving the synthetic activity of this cell cluster that highly expresses col2A1 might be a potential therapeutic strategy for retaining the fibrillar collagen network of the discs during the process of IDD. In addition, the genes of C-type lectin domain family 3 member A (CLEC3A), Collagen alpha-2(XI) chain (COL11A2), and fibroblast growth factor 1 (FGF1) in this Fc NPC cell cluster are highly expressed. Interestingly, the gene FGF1, expressed by the cell cluster Fc NPC, showed an increasing trend with the progression of IDD, suggesting that it plays a crucial role in the evolution of the NP cell fibrosis. Surprisingly, the Gene Ontology (GO) analysis showed that the cell clusters in the late stage of IDD were closely correlated to the cellular stress response, including inflammatory and endoplasmic reticulum (ER) stress response. It is speculated that these stress activities might be the main contributors accelerating IDD process in the late stage.

Although the intervertebral disc has been identified as an immune-privileged organ with no access to systemic circulation in the past few decades (Takada et al., 2002), structural deficits in the NP and annulus fibrosus (AF) and formation of tears and clefts and, in some instances, herniation allow immune cell activation and infiltration, which enhance aggrecan and collagen degradation (Risbud and Shapiro, 2014). An increasing number of studies have verified that IDD is thought to be mediated by the abnormal production of pro-inflammatory molecules secreted by both the NP and AF cells as well as macrophages, T cells, and neutrophils. Shamji et al. (2010) and Kokubo et al. (2008) reported that in herniated discs, along with the invading blood vessels, there was infiltration of CD68^+^ macrophages, neutrophils, and T cells (CD4^+^, CD8^+^). In our study, we identified four clusters of immune cells: M1 macrophage (Mac M1), M2 macrophage (Mac M2), T cells, neutrophils (Neu), and myeloid progenitor cells (Mye Pro C). The scRNA-seq in our study showed that apolipoprotein (APOE) was highly expressed in the M1 macrophage cluster that highly expressed CD163^+^, suggesting that APOE might play a crucial role in the cellular activity of M1 macrophage. Another interesting finding is that the proportion of M2 macrophage cluster drops with the progression of IDD, followed by the increasing trend of the proportion of M1 macrophage. This suggests that macrophage polarization might play an important role in amplifying the inflammatory cascade in the process of IDD. The cellular interaction analysis showed that nuclear factor kappa B (NF-κB) played an important role in the macrophage polarization. It has been reported that the NF-κB pathway was one of the most important signaling pathways that mediated inflammation in the IDD process (Wuertz et al., 2012; Hu et al., 2020). However, the deep mechanisms remains unclear. In this study, scRNA-seq analysis showed that MIFs were highly expressed in the cell clusters of MAC M1, Pro NPC, and IR NPC. Further cellular interaction analysis showed that MIF signaling pathway was highly activated between MAC M1 and Pro NPC. MIF is a protein coding gene, which plays a role in the regulation of macrophage function in host defense (Calandra and Roger, 2003). These results predicted that NF-κB-mediated macrophage polarization might influence the activity of Pro NPC *via* inducing inflammatory response through the MIF signaling pathway. In our scRNA-seq results, the cell cluster of neutrophils highly expressed gene S100A8, which has been considered as a diagnostic and therapeutic target in inflammation-associated disease (Wang et al., 2018). Sott et al. reported that S100A8 could mediate neutrophil accumulation by upregulating the integrin molecule CD11b (Scott et al., 2020). These interesting results suggest that targeting inflammation-dependent responses, such as macrophage amplification and neutrophil recruitment, could be a better therapeutic strategy for patients with DDD.

Cell metabolism, including anabolic and catabolic activities, is important for maintaining disc integrity and health by producing ECM components and chemicals responsible for breaking down the matrix, and even cell viability (Grunhagen et al., 2006; Wang et al., 2013). These anabolic and catabolic processes are highly energy demanding. It has been reported that the disc microenvironments, i.e., inflammatory cascade and mechanical stress, contribute significantly to the cell metabolic activity and senescence (Silagi et al., 2018). Therefore, a deeper mechanistic understanding of metabolic regulating network for cellular and cell–ECM interaction is of importance (Bedore et al., 2014; Wang et al., 2020). In this study, we identified two new populations of NP cells (Met NPC and IR NPC) that were closely related to metabolism and inflammatory response, respectively. Further analysis showed that matrix remodeling-associated protein 5 (MXRA5) and Dickkopf-related protein 3 (DKK3) were highly expressed in the Met NPC. MXRA5, also known as adhesion protein with leucine-rich repeats and immunoglobulin domains related to perlecan (Adlican), is a protein of unknown function belonging to the MXRA gene family. Poveda et al. reported that MXRA5 played a potential role in tissue injury and fibrosis. They identified that MXRA5 could be upregulated by transforming growth factor-β (TGFβ1) and functioned as an anti-inflammatory and anti-fibrotic molecule (Poveda et al., 2017). The cell-communication analysis showed that TGFβ signaling was the main pathway in the interaction between macrophages and Met PNC, which predicted that the MXRA5/TGFβ1 axis might be the potential target for immunometabolism intervention during the process of IDD.

Previous studies have reported that the degenerative changes in the intervertebral discs, such as composition of ECM, loss of disc cells, and proteoglycan and water content, were suggested to be the consequence of an upregulation of catabolic matrix metalloproteinases (MMPs) (Weiler et al., 2002). Consistent with previous studies, matrix metalloproteinase-3 (MMP3) was also found to be highly expressed in the inflammatory response NPC cluster (IR NPC), which accounted for a highly proportion of NP cells in the late stage of IDD. This is also consistent with our previous study that has demonstrated that inflammation was one of the main contributors to IDD (Molinos et al., 2015; Wei et al., 2015; Wei et al., 2019; Sun et al., 2021). Further analysis showed that the gene thioredoxin-interacting protein (TXNIP), early growth response 1 (ERG1), and fibroblast growth factor 1 (FGF1) were also highly expressed in this cluster. It has been reported that FGF1 was extensively related to tissue fibrosis (MacKenzie et al., 2015; Shimbori et al., 2016). In this study, highly expressed FGF1 in the IR NPC cluster might predict the close relationship between fibrosis and inflammation in the late stage of IDD. However, the specific mechanisms are warranted for further study. Functional enrichment analysis suggested that endoplasmic reticulum stress was activated in the IR NPC cluster. Consistent with this analysis, ERG1 was highly expressed in this cluster. A previous study reported that the extracellular regulated kinase (ERK) arm of the mitogen-activated protein kinase (MAPK) signaling pathway could mediate endoplasmic reticulum stress-induced ERG1 (Zou and Liu, 2021). These results provide new clues for gene intervention related to the progression of IDD.

Several limitations of this study should be acknowledged. First, the normal NP tissues were absent in this study due to the difficult collection of human samples in clinic. Therefore, the results might not fully identify the cell populations at single-cell resolution. In addition, to be honest, it is a real tough work to isolate adequate cells from highly degenerated discs. Thus, the most degenerated disc (grading Ⅴ based on the Pfirrmann grading system) was also not included in this study. Lastly, we did not verify most of the results using large-scale clinical samples and data. Hence, further studies are warranted in the near future, to overcome the limitations mentioned above.

In conclusion, our scRNA-seq results allowed for identification of NP cell populations at single-cell resolution and at the relatively whole-transcriptome scale, followed by the paths of cellular communications, discriminative markers, and transcription factors in relation to specific cell subsets (i.e., NP cells and immune cells), especially the interaction between macrophage polarization and the metabolism of NP progenitor cells, which might play a crucial role in the progression of IDD. These new findings open new possibilities for providing diagnostic and therapeutic strategies for IDD-related bioremediation and healthcare.

## Materials and Methods

### NP Samples

NP tissues were obtained from three patients diagnosed with lumbar disc herniation at the levels of L4–L5 or L5–S1, who were undergoing discectomy. All the patients were males and the age was 23, 32, and 44 years old, respectively. All participants included were confirmed with MRI images before surgery. Pfirrmann grading was used to assess the degree of degeneration (Pfirrmann et al., 2001). This study was approved by the Ethics Committee of Zhongshan Hospital, Fudan University (B2019-178). All the patients signed informed consent forms before participation. The intervertebral disc tissue resected during the operation was transported immediately to the laboratory under 4°C cold storage for single-cell suspension preparation.

### Isolation of Human NP Cells

When the NP samples were delivered, they were washed at least 3 times in sterile PBS buffer to wash away blood and prevent contamination. The NP tissues were then cut into 1 mm^3^ pieces in PBS buffer, and digested with 1 mg/ml type II collagenase (Gibco, United States) at 37°C in an atmosphere of 5% CO_2_ for 2–4 h until the tissue dissolved into a single-cell suspension. The isolated cells were filtered using 40-μm nylon filters (BD, United States) and washed twice with sterile phosphate-buffered saline containing 5% fetal bovine serum (Gibco) to remove cell fragments. All steps were performed on ice to preserve cell viability. Cell activity was determined by trypan blue staining; only samples with a viability of >80% were used in subsequent experiments. The cell density was then adjusted to 10^6^/ml for scRNA-seq.

### Library Construction for 10X scRNA-Seq

The libraries were prepared with Chromium Single cell 3′ Reagent v3 Kits (10× Genomics, United States) according to the manufacturer’s protocol. Each single-cell suspension was mixed with primers, enzymes, and the gel beads containing barcode information, and then loaded on a Chromium Single Cell Controller (10× Genomics) to generate single-cell gel beads in emulsions (GEMs). Each gel bead was bonded to one single cell and then wrapped with an oil surfactant. After generating the GEMs, reverse transcription was performed using barcoded full-length cDNA followed by the disruption of emulsions using the recovery agent and cDNA clean up with DynaBeads Myone Silane Beads (Thermo Fisher Scientific, United States). cDNA was then amplified by PCR with an appropriate number of cycles and thermal conditions that depended on the recovery cells. Subsequently, the amplified cDNA was fragmented, end-repaired, A-tailed, ligated to an index adaptor, and subjected to library amplification. The cDNA library was sequenced on a NovaSeq 6000 sequencer (Illumina, United States). These procedures were performed by OE Biotech, Inc., Shanghai, China. Raw data can be found in online repositories (https://www.biosino.org/node/search), and the accession number is oep001692.

### snRNA-Seq Data Analysis

#### Alignment

Raw reads were aligned to the human genome (hg38), and gene expression matrices were generated for each sample by the cell ranger (v6.0.1) count function with default parameters.

### Cell Clustering and Cell-type Annotation

The R package Seurat (v4.0.5) was used to cluster the cells in the merged matrix data. Cells with <200 transcripts and >5,000 transcripts detected were filtered out as low-quality cells. From the filtered cells, the gene expression matrices were normalized to the total UMI counts per cell and transformed to the natural log scale. To correct the batch effects, we integrated different samples using the R package Harmony. The FindVariableFeatures function was used to obtain the top 2,000 highly variable genes (HVGs) of each sample. The default dimensions parameters (1:20) were used for the anchor-weighting procedure. The integrated dataset on all cells was then used to scale and center the genes, and compute the principal components (PCs). After PCA to reduce dimensionality and build k-nearest neighbor graphs (*k* = 10) of the cells with the function FindNeighbors based on the Euclidean distance in the 50-dimensional PC space, the main cell cluster was identified using the Louvain-Jaccard graph-based method. For classifying all filtered cells, the clustering parameter resolution was set to be 2.0 with the function FindClusters in Seurat. Next, the function RunUMAP with dimensions parameters (1:30) in Seurat was used to reduce high dimension into two dimensions (2D) for visualization. Lastly, we run the Seurat FindAllMarkers function to identify the genes specifically expressed in each cluster. The significance of the differences in gene expression was determined using the Wilcoxon rank sum test with Bonferroni correction, and cell types were manually annotated based on the cluster markers. A marker-cluster heatmap was generated with the R pheatmap (v1.0.12) package, and the marker genes of each cluster were used to perform GO enrichment analysis with clusterProfiler (v3.14.3) R package.

### DEGs Analysis

Differentially expressed genes (DEGs) testing of each cell type was performed using the FindMarkers function in Seurat. The significance of the differences in gene expression was determined using the Wilcoxon rank sum test with Bonferroni correction. The differences in genes of the two groups in each subcluster were determined based on following criteria: (1) expressed in more than 10% of the cells within either group or both groups; (2) |log_2_FC| > 0.25; and (3) Wilcoxon rank sum test adjusted *p*-value < 0.05.

### GO Enrichment Analysis

Enrichment scores (*p*-values) for selected numbers of GO annotations were calculated by clusterProfiler (v3.14.3) R package with a hyper-geometrical statistical test with a threshold of 0.05, and the Benjamini-Hochberg method was used to estimate the false discovery rate (FDR). Enrichment was calculated for the input DEGs in subcluster. The background in human data was all the genes listed in the database of org.Hs.eg.db. Lastly, the barplot function was run for visualization.

### Pseudotime Trajectory Construction

The trajectory analysis was performed using the Monocle2 R package (v2.14.0) to reveal cell state transitions in NP cell clusters and immune cell clusters. The data were normalized by the estimateSizeFactors and estimateDispersions functions with the default parameters. We used the differentially expressed genes (DEGs) to sort the cells into pseudo-time order. Dimensional reduction and cell ordering were performed using the DDRTree method and the orderCells function. Lastly, the plot_cell_trajectory function was run for visualization.

### Cell–Cell Ligand–Receptor Interaction Analysis

CellChat (v1.1.3) was applied for ligand–receptor analysis. The normalized counts and cell-type annotations for each cell were imputed into CellChat to determine the potential ligand–receptor pairs. Interaction pairs with *p* value > 0.05 were filtered out from further analysis. The interaction of Macphage and NP cells was analyzed. Selected specific pairs were plotted in CellChat with default parameters.

## Data Availability

The datasets presented in this study can be found in online repositories. The names of the repository/repositories and accession number(s) can be found below: NGDC Bioproject, accession no: PRJCA007656.
